# Supplementary material for: Sorting at embryonic boundaries requires high heterotypic interfacial tension
Source: Nat Commun. 2017 Jul 31;8:157. doi: 10.1038/s41467-017-00146-x (PMC5537356; doi:10.1038/s41467-017-00146-x)
Supplement: Supplementary file 2 — Supplementary Software 1 [file 41467_2017_146_MOESM2_ESM.zip › potts-code-guide-v2.pdf]

Potts Model Code Guide

Getting Started

Getting started

- Install the **Java compiler (javac)**
  - If you’re on a Mac or you have Matlab installed, it is already installed
  - To check that it’s installed, open Terminal (mac) or Command Prompt (Windows and type) type:
    `javac -version`

  - If you get an error instead of the version number (like below), it’s probably not installed or not linked properly.

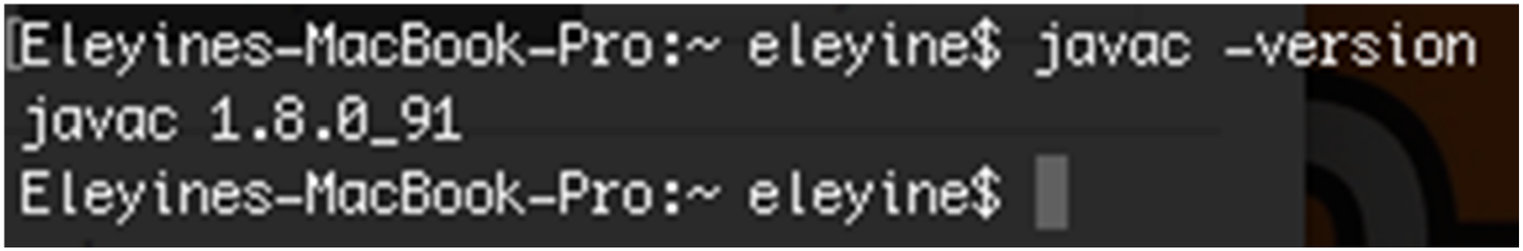

Getting started

- Install **Eclipse** (from https://eclipse.org)
  - It’s a useful Java IDE (Integrated Development Environment)
  - Helps to navigate code and inspect classes

Running a simulation

Running a simulation

- **Open the console** (Terminal on Mac, Command Prompt on Windows)

- Go to the folder **ExecutablePottsModel** containing the file **PottsModel.jar**

- Run the following command:

java -Xmx512m -jar PottsModel.jar -config config/graner-glazier.txt -lattice mtx/RandomA.txt -name simulation -mcs 200

- Example:

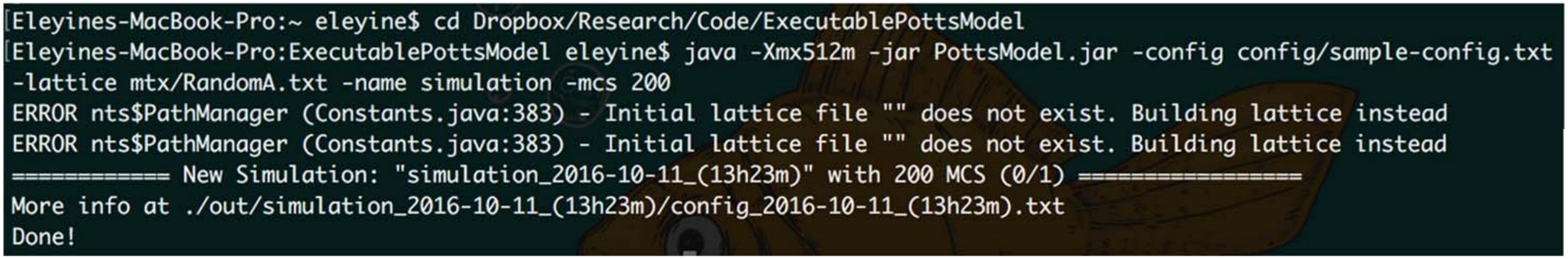

Command line skeleton

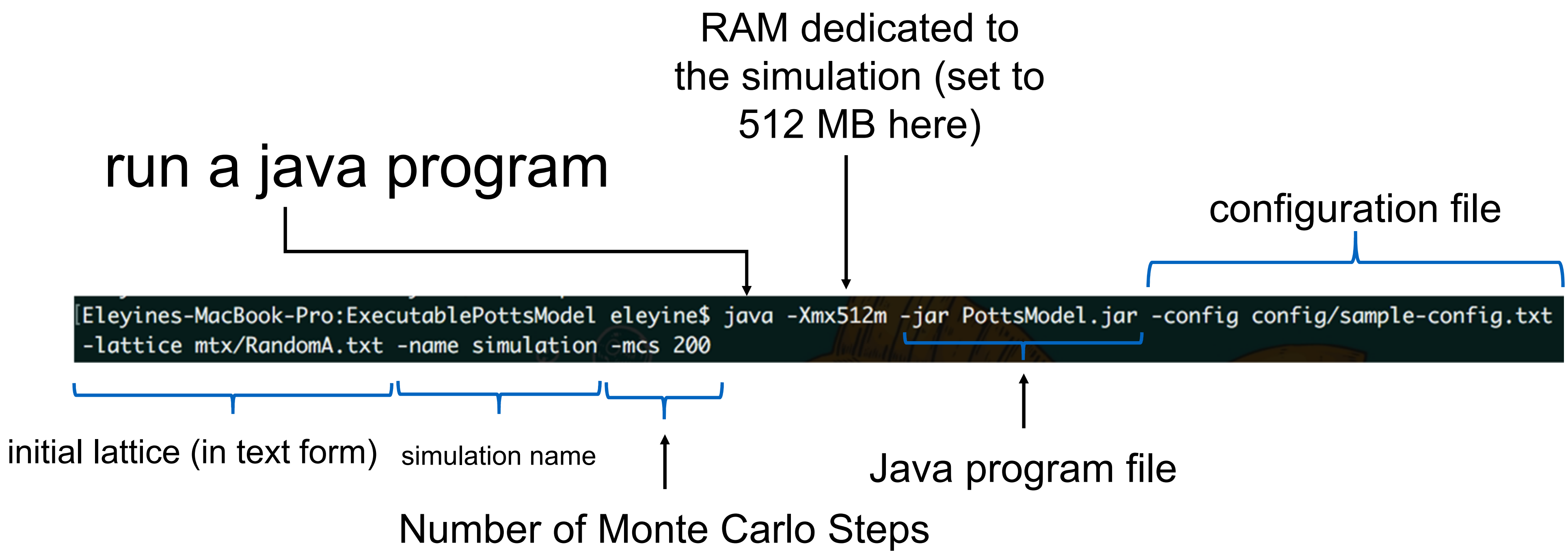

A **flag** starts with '-' and corresponds to a configurable simulation option. Spelling (including upper/lowercase) must be respected.

<u>Note</u>:

- Some flags take arguments
  ex: -config <textfile> where <textfile> = the path to the configuration file starting from the current directory
- Some flags are standalone
  ex: -graphic

Available flags

- **-dir <Output Directory>**
- **-name <SimulationName>**
- **-ee <Ecto-Ecto Energy Value>**
- **-mm <Meso-Meso Energy Value>**
- **-em <Ecto-Meso Energy Value>**
- **-config <SimulationConfigFile>**
- **-mcs <NumberOfMCS>**
- **-lattice <InitialLatticeFile>**
- **-log <LogLevel>** (<LogLevel> can be 0, 1, 2, 3 or 3)
  where 0 = OFF; 1 = Error; 2 = Info; 3 = Debug; 4 = Trace)
- **--noauto**

  if included the simulation will pause at each Monte Carlo Step and wait for user input to continue to the next step
- **--graphic**

  if included, a live graphical output of the simulation will be displayed (slower)
- **--verbose**

  if included, the log level will be set to 3 (Debug)
- **--nosnap**

  if included, no snapshots will be saved

Eclipse

- It is easier to browse the source code using **Eclipse**, for installation instructions, visit: http://www.eclipse.org/downloads/packages/eclipse-ide-java-developers/neon3
- To import the project, do: File.. Import...Existing project... and select **SrcPottsModel**

Graphical Interface

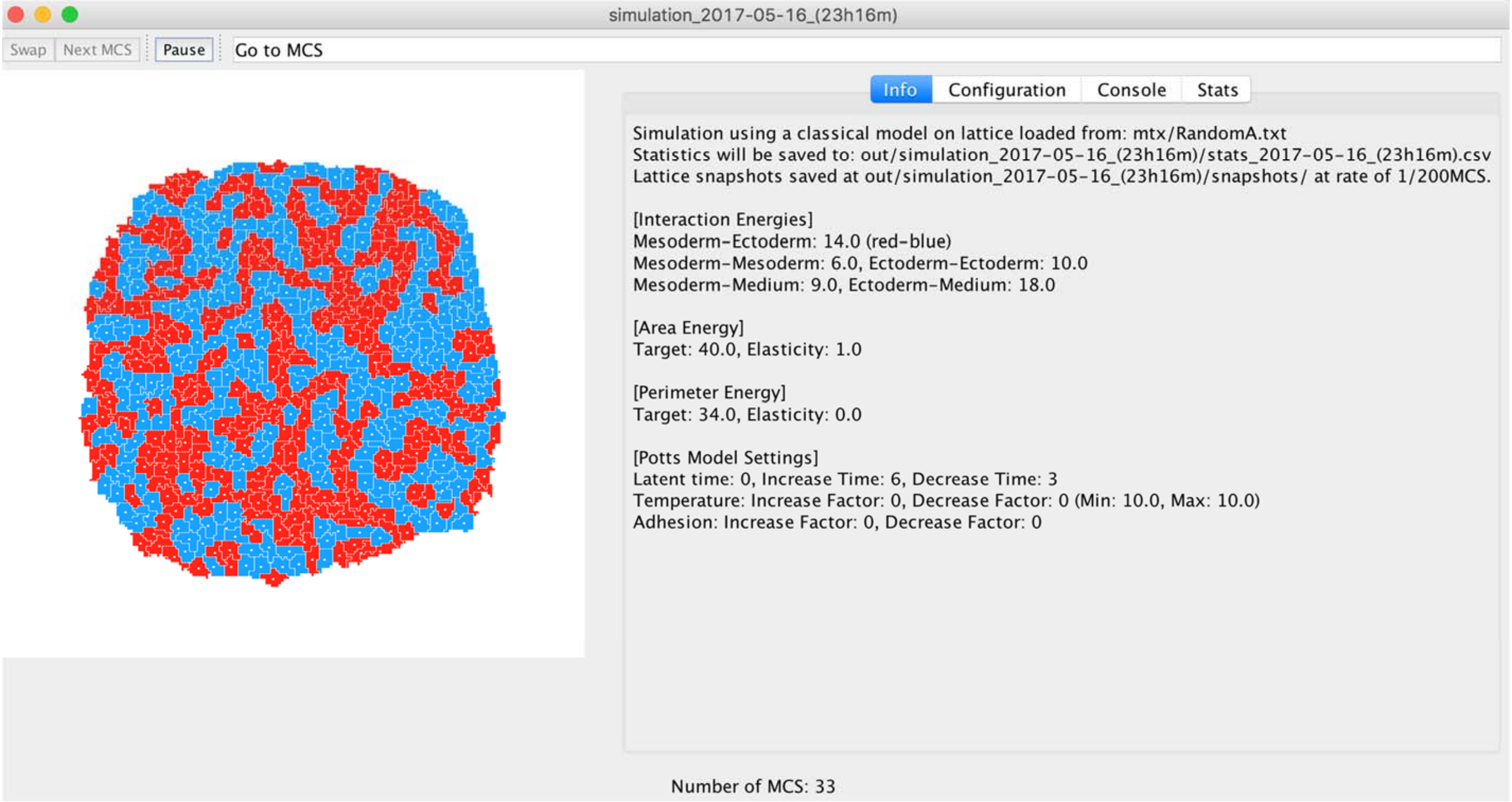

Graphical Interface

Advantages:

- Watch simulation graphical output in real time
- Change parameters and observe changes in real time
- Real-time time series for selected statistics
- Possible to pause and play
- Target specific pixel or cell to consult their parameters

Disadvantage:

- Slower

Graphical interface

- Using the <u>command line</u>, add the ***--graphic*** flag to the command line to see the graphical interface. For example:

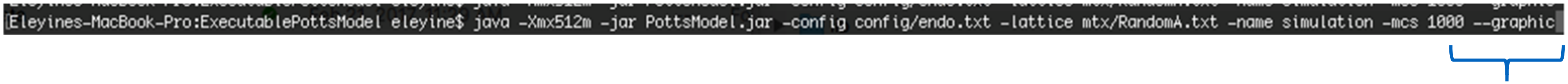

- Using <u>Eclipse</u>, select a Simulation file and click on the ”Run” button: *see next slide*

1. select a simulation file (in this case, *DefaultSimulation.java*)

2. click this button to run the simulation

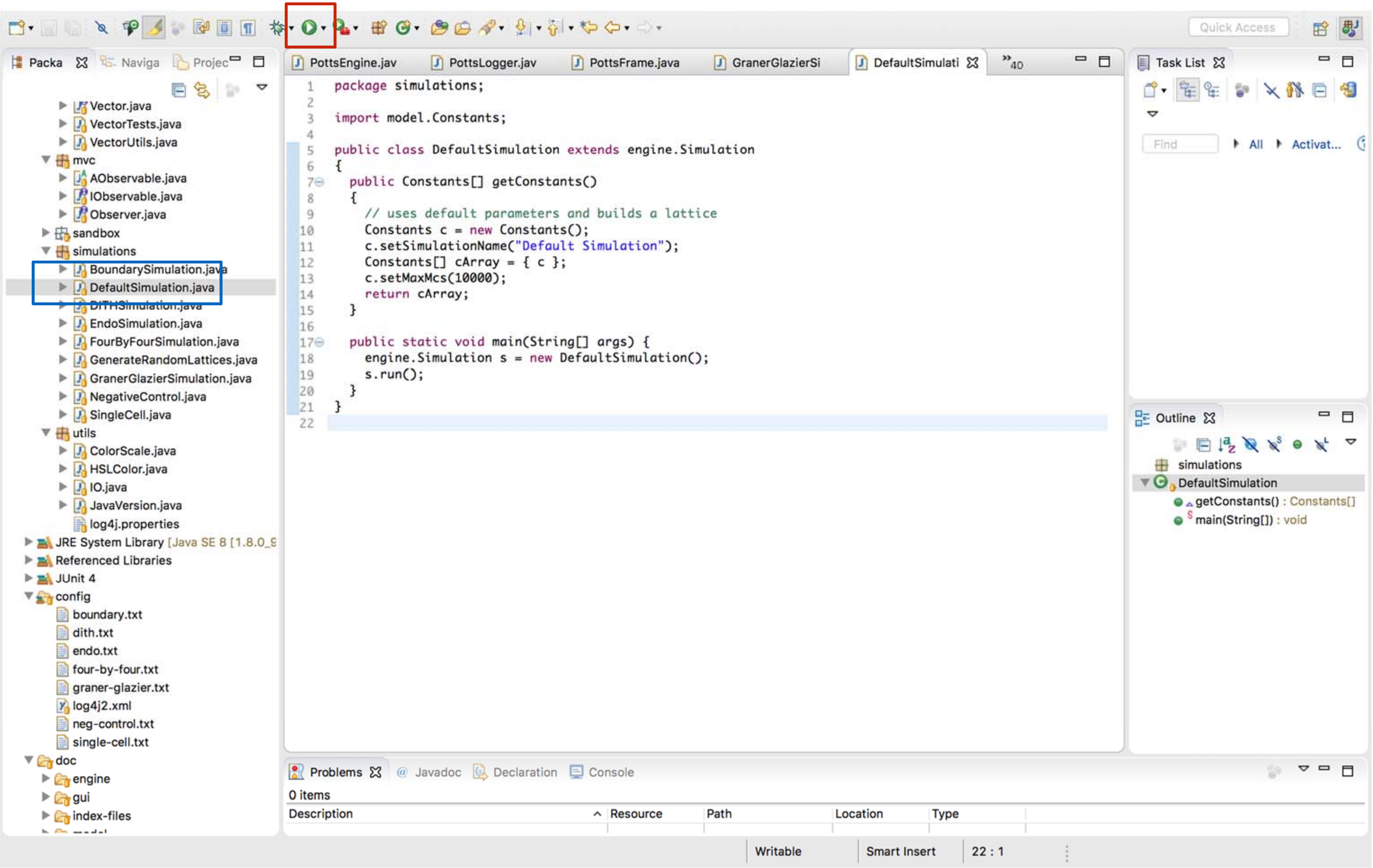

Graphical interface

- **Left-click** on a **pixel** gives you attributes of that pixel in the console
  Right-click on a **cell** gives you attributes of that cell in the console

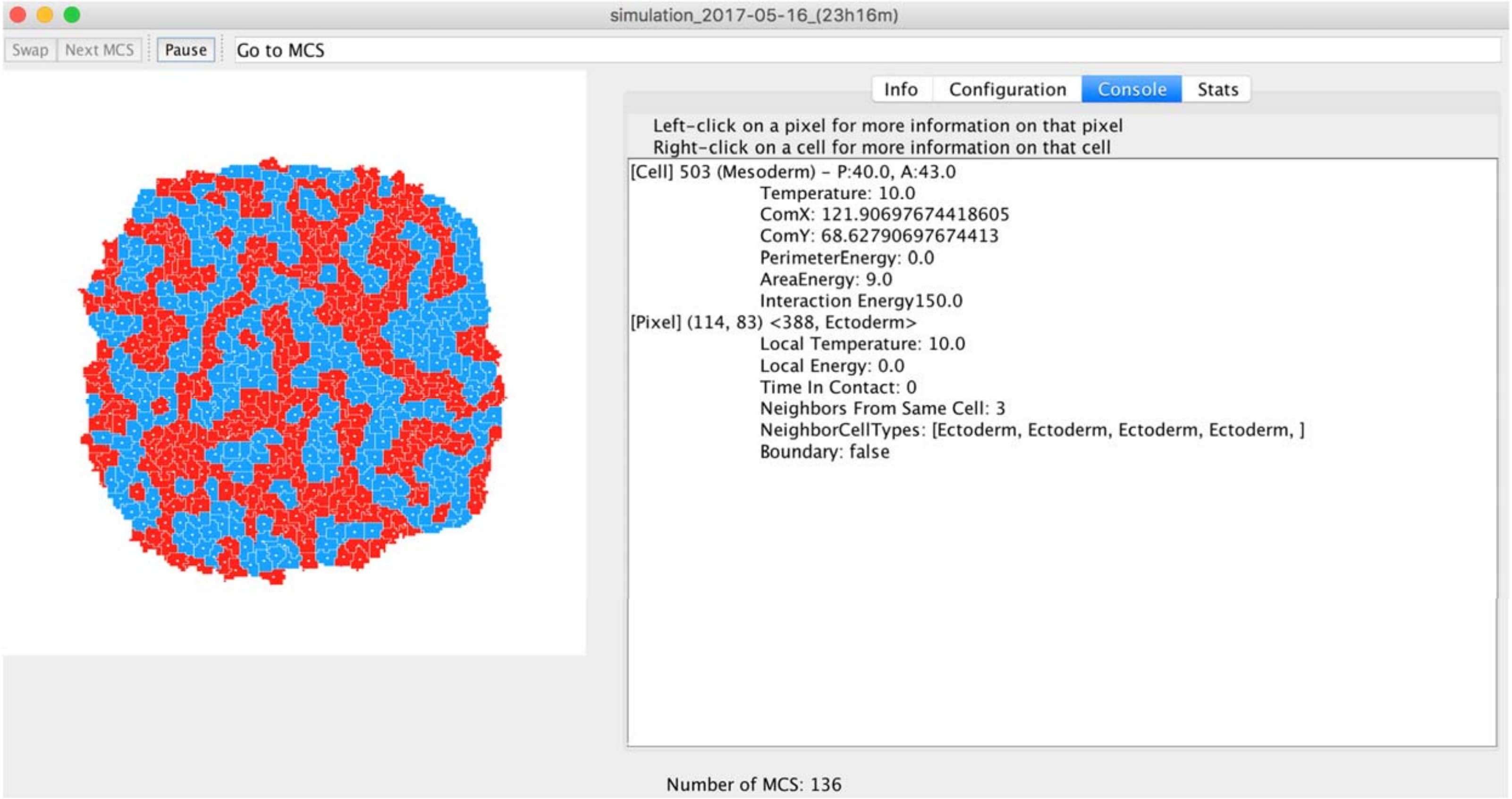

Graphical interface

- Observe statistics in real time

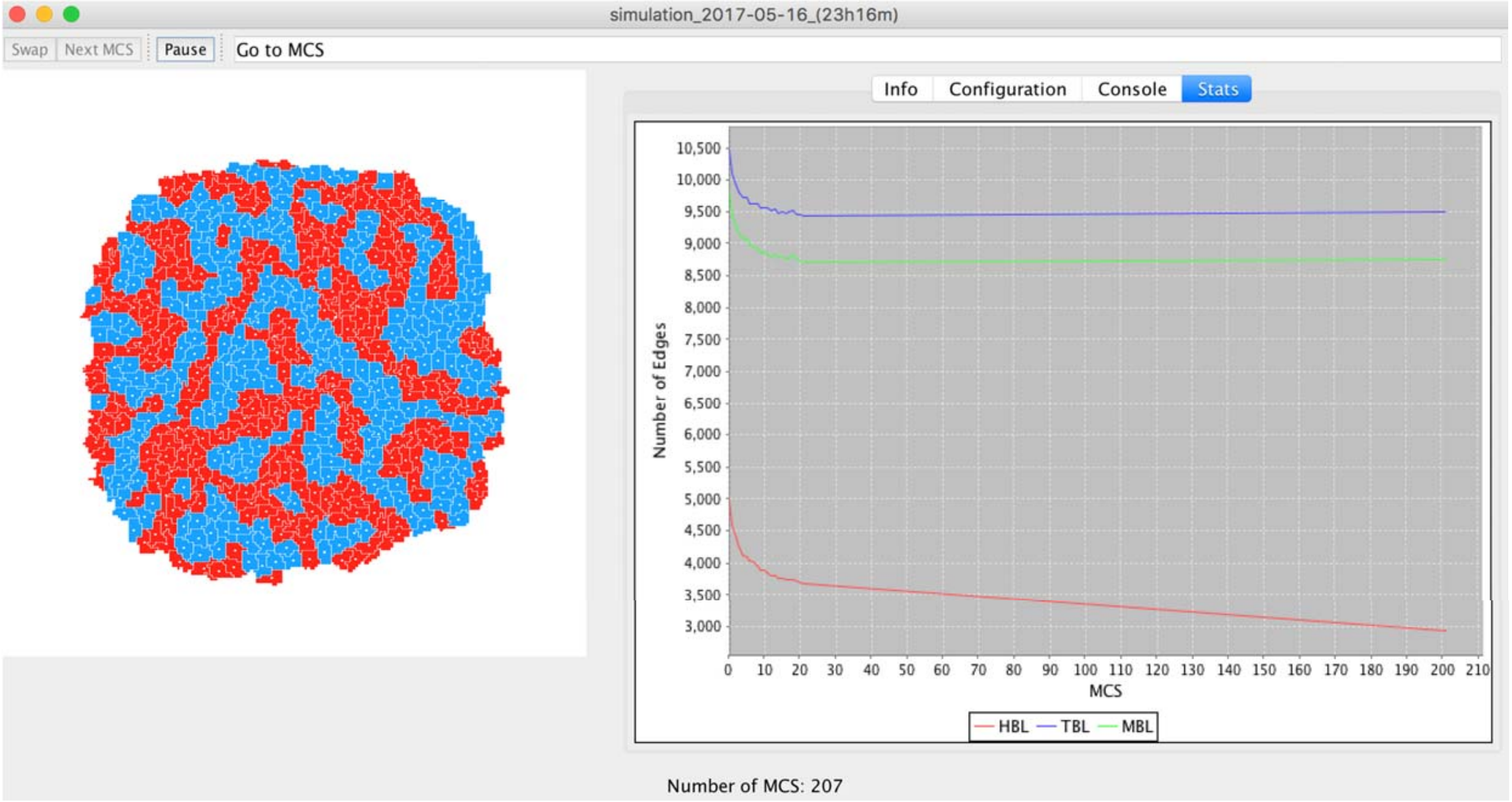

Graphical interface

- Update parameters and observe changes in real time

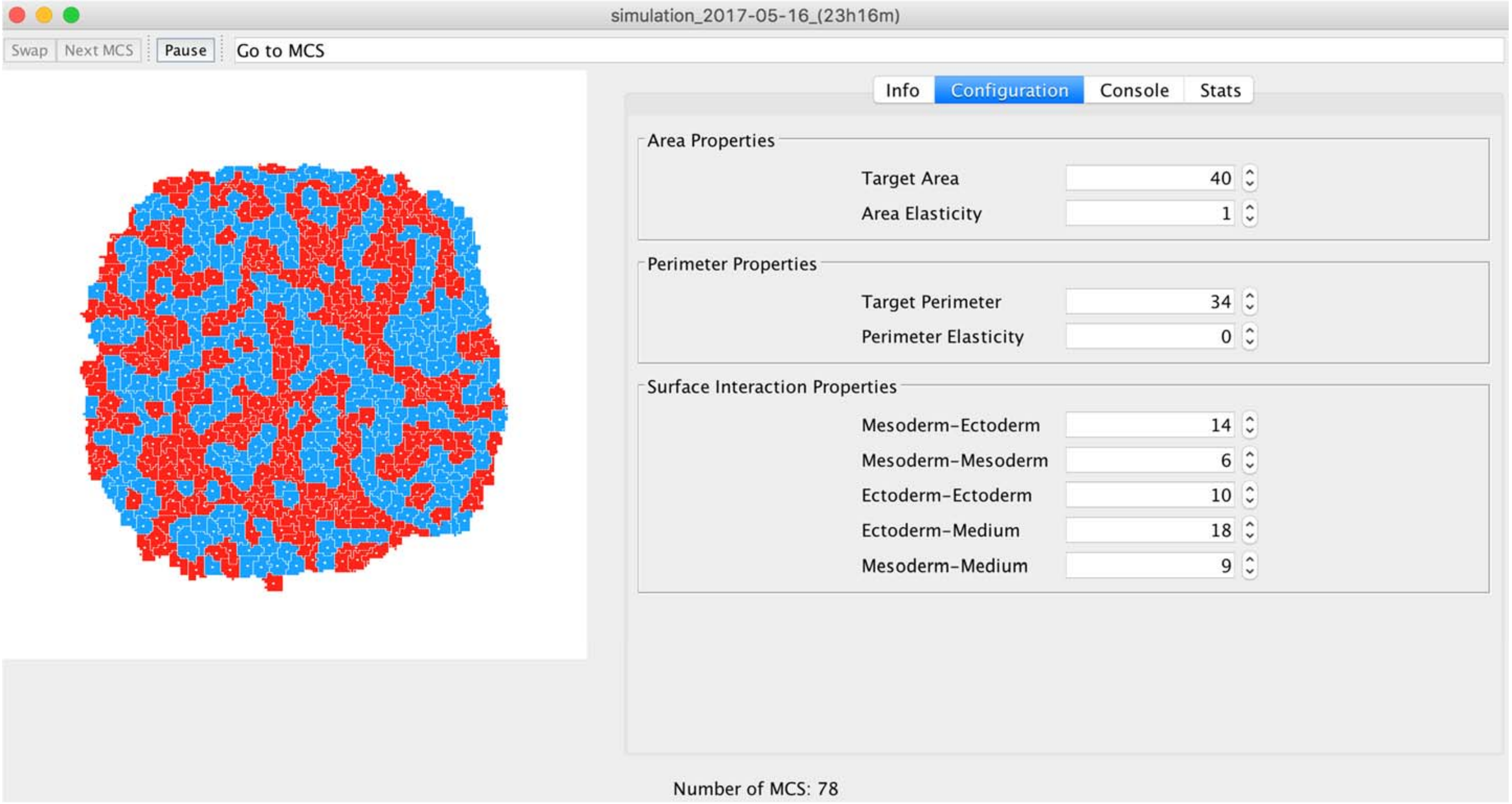

Configuration file Options

More simulation options can be specified in the config file. Note however that the options specified in the command line will override the corresponding option in the config file.

```
NAME = Default Simulation

# Simulation Parameters
PIXEL_TYPE = Square
NUMBER_MCS = 30000
NUMBER_NEIGHBOURS = 4
DEBUG_MODE = false
MODEL = Classical

# lattice
BUILD_LATTICE = false
FILE_INITIAL_LATTICE =
config/mtx/RandomA.txt

# stats
SAVE_SNAPSHOT = true
FREQ_SNAPSHOT = 200
SAVE_CSV = true
FREQ_CSV = 200
SAVE_LATTICE = true
FREQ_LATTICE = 500

# type of stats
TRACK_HBL = true
TRACK_ENERGIES = false
TRACK_DISPERSION = false
TRACK_AREA = false
TRACK_PERIMETER = false
TRACK_IQ = false
TRACK_NN = false
TRACK_SHAPE = false
TRACK_COORD = true
TRACK_HMD = false
NN_NUM = 3

# GUI
PIXEL_SIZE=2
SCALE_PIXEL_SIZE = false

# Temperature variation parameters
LATENT_TIME = 0
INCREASE_TIME = 6
DECREASE_TIME = 3
INCREASE_FACTOR_TEMPERATURE = 0
DECREASE_FACTOR_TEMPERATURE = 0
INCREASE_FACTOR_ADHESION = 0
DECREASE_FACTOR_ADHESION = 0

# Heat Map
HEATMAP_MODE=false
HEATMAP_PARAM=angle

# Interaction energies
# MESO = LIGHT, ECTO = DARK
WITHIN_CELL = 0
MESO_MEDIUM = 25
ECTO_MEDIUM = 25
MESO_MESO = 10
ECTO_ECTO = 10
MESO_ECTO = 14

# Cell default parameters
MIN_TEMPERATURE = 10
MAX_TEMPERATURE = 10
AREA_ELASTICITY = 1
PERIMETER_ELASTICITY = 0
TARGET_AREA = 40
TARGET_PERIMETER = 34
PROTRUSION_ELASTICITY=0
```

Additional info

- 1 option / line
- anything after # is ignored (used for internal comments)
- empty lines are ignored
- case/spelling must be respected

Acronyms:

- MCS = Monte Carlo Step
- CSV = comma-separated file
- HBL = heterotypic boundary length
- IQ = isoperimetric quotient
- NN = nearest neighbor
- HMD = homotypic minimal distance
- COORD = coordinates
- GUI = graphical user interface
- FREQ_SNAPSHOT = frequency of snapshots (in the example, there’s a snapshot for every 200 MCS)
- FREQ_LATTICE = same as the above for the lattice in text format
- FREQ_CSV = same as the above for statistics in CSV format

Programmatically changing configuration options

- It's possible to create a Simulation object and define the simulation options within the code itself. To do that, your class must be a subclass of the **engine.Simulation abstract class**.

- Example simulation classes can be found under /src/simulations/

- You must implement the getConstants() method and return a Constants instance. The Constants object can be customised as desired (see **model.Constants**)

- Example:

```java
package simulations;

import model.Constants;

public class SingleCell extends Simulation {


    public static void main(String[] args) {
        SingleCell s = new SingleCell();
        s.run();
    }

    @Override
    public Constants[] getConstants() {
        Constants c = new Constants("config/ref/singleRealisticCellConfig.txt");
        Constants[] cArray = { c };
//      c.setMaxMcs(5);
        return cArray;
    }

}
```

Simulation output

If the ”--graphic” flag is not included, the simulation’s output can be found under **out/<SimulationName>_<Timestamp>**

For example, a simulation named ”simulation” (as specified in the command line flag or in the config file) and ran on Oct 11 2016 at 13h23 will be found in the folder: **simulation_2016-10-11_(13h23m)**

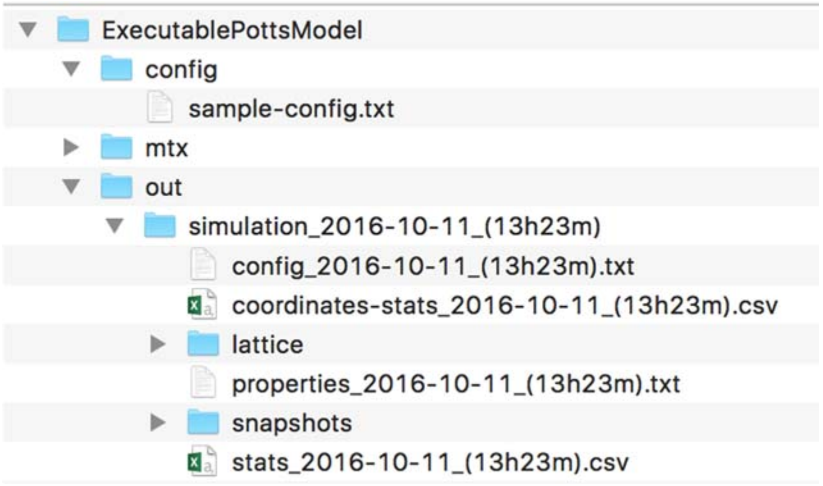

Available files:

- A configuration summary specific to this simulation
- Coordinate statistics (if TRACK_COORD was set to true)
- Lattice states in text format
- Simulation properties (less readable than the config summary) = the Constants object used for this simulation
- Snapshots in .png format
- Statistics for this simulation in CSV format

Code Overview

engine.PottsEngine

- Found under /src/engine
- Class describing core loop functionality of the PottsModel simulation. It needs to be passed a Constants object to initialize the correct Lattice (e.g. checkered, boundary, custom) and the correct energy computation model.

model.EnergyManager

- Found under /src/model
- Associates energy functions with simulation models. The CLASSICAL model for example uses AreaEnergyFunction, PerimeterEnergyFunction, and PixelInteractionEnergyFunction.

model.EnergyFunction

- Found under /src/model
- All energy functions used by EnergyManager must extend this abstract class and implement several methods including getDeltaEnergy(Pixel source, Pixel target) which must return the energy difference associated with this particular energy function resulting from copying the source pixel onto the target pixel.

model.Pixel

- Found under /src/model
- Pixels are instantiated only once when a Lattice is created (by PottsEngine) and have final (immutable) x and y positions. Pixels change the cells they belong to during the simulation. This class is not to be confused with graphical pixels (see PixelDisplay for handling graphical representation of Pixels).
- Pixels have a PositionManager that defines which Pixels are considered neighbors (useful when dealing with Square pixels vs Hexagon pixels, which also have (x,y) positions). What differentiates a square pixel from a hexagon pixel is its PositionManager and its PixelDisplay only.

model.Cell

- Found under /src/model
- Cells are also instantiated once. They have a mutable list of Pixels. This class makes sure that perimeter and area are computed correctly and keep track of various things like their cell type or their center of mass.

More documentation is available at:
SrcPottsModel/doc/index.html

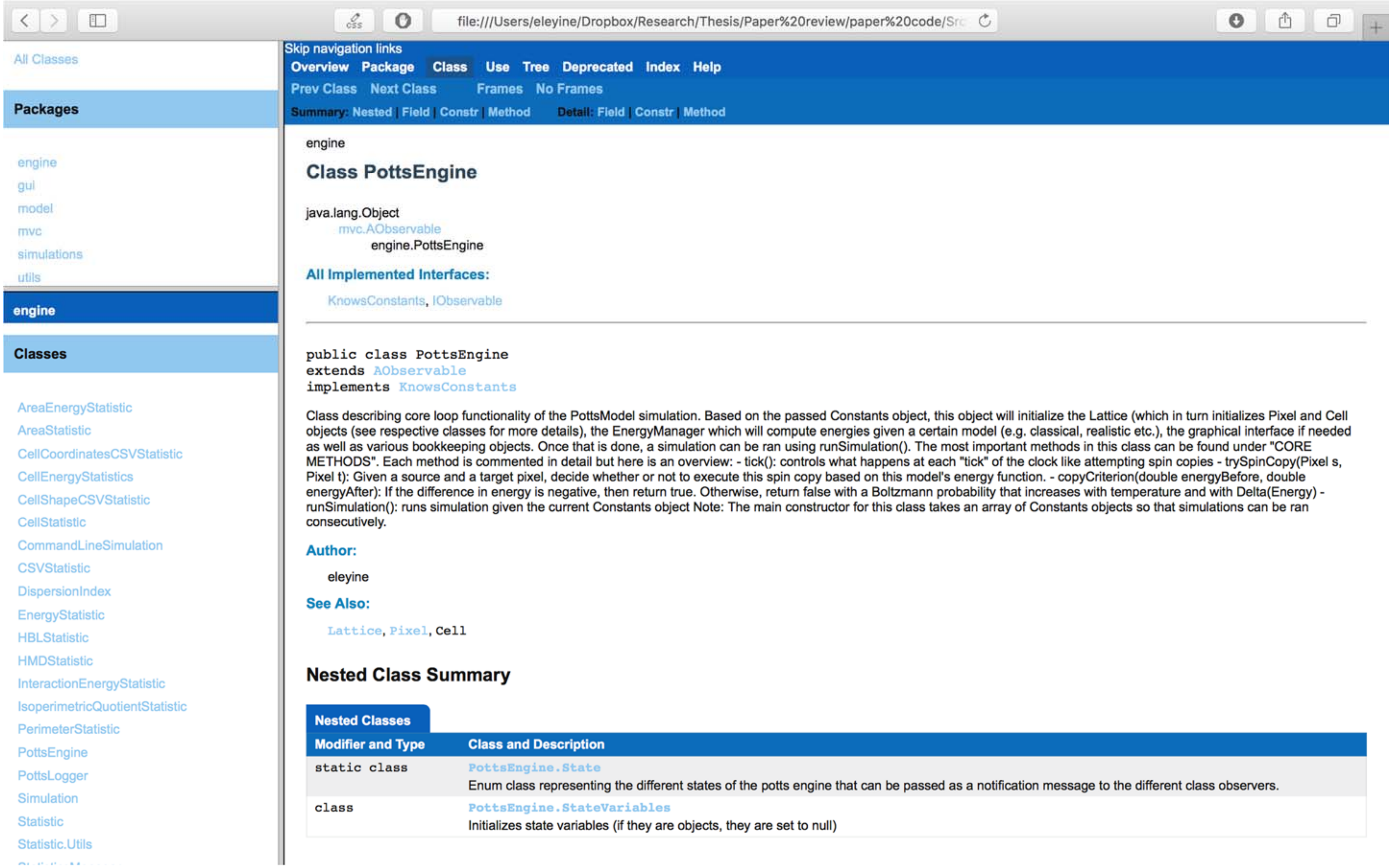

Miscellanous

Observer Pattern

- Some classes (the PottsEngine mainly) send notifications when certain events occur. For example, the PottsEngine sends a newMCS notification when a new Monte Carlo Step begins. This allows Observer classes like the GUI classes or Statistic trackers to perform their own actions in response to these events. This coding pattern is known as the Observer pattern (there's tons of info on it online). The most important things to know are:

- each Observer must implement the mvc.Observer interface and implement the single method update(Notification n) that determines what an observer should do upon notification that the observed object has changed.

- each Observable/Observed class must extend the mvc.AObservable abstract class which handles adding/removing Observers and notifying all Observers when needed via notifyObservers(Notification n)

Eclipse tips and tricks

- Hovering over a given method or object will show documentation for that method/object. If you hover a little longer, an icon with a yellow arrow will appear. Clicking on that icon will take you to that method's declaration or the object's class source code.
- When you add the dot operator next to an Object variable, Eclipse will give you a list of possible instance variables and methods to use.
- Make sure you enable the outline view on the right to get an overview of instance variables and methods for the current class, along with their visibility (private/protected/public)
